# Selenium Nanoparticles (SeNPs) Immunomodulation Is More Than Redox Improvement: Serum Proteomics and Transcriptomic Analyses

**DOI:** 10.3390/antiox11050964

**Published:** 2022-05-13

**Authors:** Ivan Fan Xia, Hang-Kin Kong, Margaret M. H. Wu, Yishan Lu, Ka-Hing Wong, Kevin W. H. Kwok

**Affiliations:** 1 Department of Applied Biology and Chemical Technology, The Hong Kong Polytechnic University, Kowloon, Hong Kong; f.xia@yale.edu (I.F.X.); hang-kin.kong@polyu.edu.hk (H.-K.K.); manhui48.wu@connect.polyu.hk (M.M.H.W.); kahing.wong@polyu.edu.hk (K.-H.W.); 2 Section of Cardiology, Department of Internal Medicine, Yale Cardiovascular Research Center, Yale University School of Medicine, New Haven, CT 06511, USA; 3 Research Institute for Future Food, The Hong Kong Polytechnic University, Kowloon, Hong Kong; 4 College of Fishery, Guangdong Ocean University, Zhanjiang 524088, China; luys@gdou.edu.cn; 5 Shenzhen Institute of Guangdong Ocean University, Shenzhen 518120, China

**Keywords:** Orbitrap tandem mass spectrometry, antioxidant, lipid metabolism, infection

## Abstract

Selenium nanoparticles (SeNPs) are a novel elemental form selenium and often reported to possess beneficial bioactivities such as anticancer, promoting bone growth and immunomodulation. Our previous study demonstrated that chitosan-stabilized SeNPs have strong activity in immunomodulation. However, the mechanism underlying the immunomodulation of SeNPs is still unknown. The aim of this study is to identify the molecular mechanisms involved in SeNP-induced immunomodulation. Using zebrafish, as a common immunological animal model with a highly conserved molecular mechanism with other vertebrates, we conducted serum proteomic and tissue transcriptome analyses on individuals fed with SeNP in healthy or disease conditions. We also compared differences between SeNPs and an exogenous antioxidant Trolox in immune activity and redox regulation. Our results suggest that the immunomodulation activity was highly related to antioxidant activity and lipid metabolism. Interestingly, the biological functions enhanced by SeNP were almost identical in the healthy and disease conditions. However, while the SeNP was suppressing ROS in healthy individuals, it promoted ROS formation during disease condition. This might be related to the defense mechanism against pathogens. SOD and NFkβ appeared to be the key molecular switch changing effect of SeNPs when individuals undergo infection, indicating the close relationship between immune and redox regulation.

## 1. Introduction

Selenium (Se) is an essential element for all animals. Common Se exists in inorganic (e.g., selenite) or organic form (selenomethionine) and plays an important role in human health through functions and activities of various selenoproteins [1]. More than 25 selenoproteins have been identified in humans and animals, including enzymes such as glutathione peroxidase (GPx), playing important roles in regulation of the immune, antioxidant and other metabolisms [2].

The health and biological effects of Se are well-known to relate to its chemical forms. Traditionally, only effects on inorganic (e.g., selenate) and organic (selenomethionine) Se species are studied. Recently, selenium nanoparticles (SeNPs), nanoparticles of elemental Se (i.e., Se0), have been reported to have many unique biological activities, such as anticancer, bone growth promotion and immunomodulation [3,4,5]. These unique biological functions benefit in part from the effects of unique properties of nanoparticles on the cell life cycle [6] and from their excellent bioavailability [4].

One of the promises of SeNPs is their ability to modulate the immune system. For example, SeNPs were found to promote activities of T-helper cells and cytokine expression to fight tumors [7]. They have also been reported to improve the systemic and mucosal immune status [8]. SeNPs have also been proposed to be used as adjuvants for vaccines as they induced a robust Th1 cytokine response [9]. SeNP is also considered as a promising Se formulation for antioxidant function regulation [10]. For example, cysteine-stabilized SeNP was shown to have a better antioxidant effect when compared with selenite or naked SeNP [11]. SeNP was effective as an antioxidant in protecting liver against acetaminophen-induced hepatic damage [12].

It is well-established that antioxidant and immune regulations are interrelated [13]. Antioxidants have been shown in much research to have anti-inflammatory effects [14]. For SeNPs, however, it is not well-established whether its effects on the immune system is related to its antioxidant properties. Moreover, up until now, most research on SeNPs has been focused on its effects on healthy individuals; its effects on sick individuals are not known. In our previous study, we found that chitosan-stabilized SeNP could promote a wide range of innate and adaptive immune responses to confer strong protection against bacteria pathogens [5]. Therefore, this SeNP can be used as a model to understand if the antioxidant and immune regulatory effects of SeNP are related in healthy and sick individuals.

Biochemical changes in serum are often used as biomarkers indicative of the overall health and immune status of an individual, including assessments of key proteins, cytokines, and metabolites [15]. The evaluation of serum proteins is a well-established laboratory approach in the diagnosis of many diseases [16,17]. For example, serum immunoglobulin levels are frequently increased during the infection of pathogens [18]. Complement components in serum are responsible for a number of reactions that help fight infections, such as the opsonization of pathogens [19]. Selenoproteins such as the selenoprotein *p* and GPx present in serum are also widely used as indicators of Se and the redox status in a given individual, respectively [20]. Therefore, variations in the levels of serum proteins can be used to reflect the health of the host and can provide insight into the physiological status of the host.

Inflammation-induced innate immune cell activities require energy. For example, T-lymphocyte proliferation is highly reliant on glycolysis and the degradation of lipids for energy through β-oxidation [21]. Macrophages reduced the inflammatory phenotype through enhancing the β-oxidation of lipids by inhibiting the activity of acetyl-CoA carboxylase (ACC) through phosphorylation [21]. A previous study reported that Se supplementation-induced ROS–lipid metabolism is highly linked with immune responses [22]. Se supplementation (e.g., deficiency or excess) is highly associated with lipid metabolism [23,24]. For example, dietary Se deficiency suppressed lipid synthesis in a pig liver and activated signaling pathway links to nutrient sensing, which led to a low body weight and deficient energy [25]. Conversely, excessive Se supplementation induced hyperglycemia and hyperinsulinemia, which are associated with the suppression of sugar metabolism and elevated lipid metabolism [26]. Tang reported that dietary Se supplementation induced reactive oxygen species (ROS), which, in turn, modulated hepatic energy metabolism (e.g., central carbon and lipid metabolism) and inflammation [25,27]. ROS regulates lipid synthesis through de novo synthesis lipid substrates such as citrate and acetyl-CoA [25]. Therefore, it is interesting to understand if SeNP plays a role in regulating lipid metabolism in relation to its immunomodulation activities. 

In this study, SeNPs were synthesized using chitosan as a stabilizer. We compare immune responses and redox conditions of the animals under healthy and disease conditions after exposed to SeNPs in the diet. Serum proteomics and transcriptome analyses were used to explore the potential mechanisms of SeNP immunomodulation. We also expose zebrafish to this SeNP via diet and contrast that with a diet containing exogenous antioxidant Trolox. We will explore the immune mechanisms following these three hypotheses: (1) SeNP and Trolox have different regulations in the immune responses and antioxidative activity, (2) SeNP changes different serum protein expressions in immune activity in healthy and disease condition and (3) the immunomodulation of SeNP might rely on Se endogenous metabolism and lipid regulation in the body.

## 2. Materials and Methods

### 2.1. Selenium Nanoparticles (SeNPs) Preparation and Characterization

Selenium nanoparticles (SeNPs) using chitosan as a stabilizer were synthesized using a controllable reduction method, as described in Xia et al. [5]. Freshly prepared ascorbic acid solution (100 mM) was added into aqueous 0.25% (*w*/*v*) chitosan solution and mixed by magnetic stirring. Drop by drop sodium selenite solution (25 mM) was added into the mixture in the dark. The mixture was then made up to 25 mL by MilliQ water (Millipore, Burlington, MA, USA) and allowed to react at room temperature for 12 h in the dark before subjected to extensive dialysis (Mw cut off: 8000).

Size distribution of the nanoparticle was measured by high-resolution transmission electron microscopy (HRTEM; JEOL 2010, Horiba EX-250, Peabody, MA, USA) and NanoSight NS300 (Malvern Instruments Ltd., Worcestershire, UK). The elemental compositions of the SeNPs were measured by energy dispersive X-ray spectroscopy (EDX) under TEM. HRTEM and selected area electron diffraction (SAEN) pattern of the SeNPs were acquired on a JEOL 2010 microspore to understand the crystal structure of the NP. The Se concentration in the SeNP stock was determined by ICP-MS (Agilent 7500, Santa Clara, CA, USA).

### 2.2. SeNPs Diet and Trolox Diet Preparation

Preparation of the SeNP diet followed the method described in Xia et al. [5]. A diet containing 10 µg/g SeNP was made by thoroughly mixing the appropriate volume of SeNP stock with 10 g of dry fish diet (Otohime B1, Campbell, CA, USA) in a Petri dish (Thermo Fisher, Waltham, MA, USA). The mixture was then freeze-dried for 48 h and passed through a 100-µm sieve to break into particle sizes suitable for zebrafish.

The total antioxidant capacity of the SeNP was determined using an antioxidant capacity assay kit (Sigma, Burlington, MA, USA). The results indicated that 1 M SeNP (1 M in Se) had a total antioxidant capacity equal to 0.349 M Trolox. The total antioxidant capacity of 10 µg/g SeNP was equal to 11 µg/g Trolox. A Trolox diet (11 µg/g) was prepared by dissolving 110 µg Trolox in 10 mL MilliQ water and mixed with 10 g base fish diet. A control diet was prepared by mixing with MilliQ water only using the same protocol. All diets were preserved in 50 mL centrifuge tubes at 4 °C in dark until the experiment. The SeNP diet was analyzed by TEM and EDX. The total Se concentrations of the experiment diets were determined by IPC-MS.

### 2.3. Fish Maintenance

Adult zebrafish (*Danio rerio*) was maintained in a 15-L tank flowthrough system at a temperature of 28 ± 0.5 °C and pH 7.0 ± 0.2 under a 14:10 light/dark cycle. Daily care and experiments carried out were under approved animal care and ethics protocols of the institution (19–20/70-ABCT-R-GRF). Fish were fed with commercial fish feed (Otohime B1, Reed Mariculture, Campbell, CA, USA) three times per day and supplemented with live brine shrimp nauplii once a day.

### 2.4. Comparison of Immunomodulation Effects with SeNP and Trolox Diet

We performed a bacterial challenge to examine the immune responses of individuals under the SeNP or Trolox diet. A total of 240 male zebrafish (~9 months) were randomly selected and acclimated in experimental tanks for 3 days before experiment. One hundred and twenty fish were fed with the SeNP diet, while another 120 fish were fed with only the base diet as the control. The fish were fed at a daily ration of 2% body weight. After 9 days of feeding, the fish in each treatment were randomly separated into two groups. The disease group contained 60 zebrafish intraperitoneally (ip) injected with 2.5 × 106 cfu of *Aeromonas hydrophila* (ATCC 7699, Manassas, VA, USA) in 10 µL using a 33-gauge needle. This protocol has been validated in our laboratory and can induce ~70% mortality in 72 h [5]. The remaining 60 fish in each diet treatment were subjected to an ip injection of 10 µL phosphate-buffered saline (PBS) as a control and are denoted as the healthy group. The survival rate of fish after the injection were recorded every 4 h until 72 h post-injection. Zebrafish that survived until the end of the experiment were euthanized to collect blood and serum for proteomic analyses. This study only used male zebrafish as the preliminary data showed that the serum from female fish contained high levels of vitellogenin in the serum and interfered with the detection of lower abundance proteins.

Immune response biomarkers (lysozyme activity, phagocytic respiratory burst activity and lymphocytes proliferation) were studied to compare these two equal antioxidant capacity diets. Zebrafish was fed with the SeNP diet, Trolox diet and the control diet at a ration of 2% body weight per day. After 9 days of exposure, 45 zebrafish from each treatment were sacrificed to collect the serum, liver, kidney and spleen, and each replicate was pooled from 5 individuals. Serum lysozyme activity, phagocytic respiratory burst activity, T-cell and B-cell proliferation were measured using previously published methods. [5].

### 2.5. Comparison of Antioxidation Effects with SeNP and Trolox Diet

The total intracellular reactive oxygen species (ROS) was measured with a commercial kit (Abcam, Cambridge, UK). For each replicate, liver samples from 5 individual zebrafish were homogenized on ice in 1 mL of PBS at pH 7.0. Homogenized samples were then incubated with 2,7-dichlorodihydrofluorescein diacetate (DCFH-DA) in a dark, humidified chamber for 20 min at 28 °C. The ROS concentration was measured on a plate on a fluorescence plate reader at Ex/Em = 485/535 nm after incubation. The ROS concentration was normalized against the protein concentration in samples. 

The total reduced GSH concentration was measured with a commercial kit (Nanjing Jiancheng Bioengineering Institute, Nanjing, China). Liver homogenates (500 µL) from 5 individuals were diluted with 1 mL of PBS and centrifuged under 4000× *g* for 10 min at 4 °C. The supernatants were incubated with 5,5-dithio-bis-(2-nitrobenzoic acid) to produce a yellow color with 5-thio-2-nitrobenzoic acid (TNB). Mixed disulphide, GSTNB (between GSH and TNB), is reduced by glutathione reductase (GR) to regenerate GSH and release TNB. The rate of TNB production is directly proportional to this reaction, which is, in turn, directly proportional to the concentration of GSH. The concentration of TNB was measured at 405–414 nm normalized by the protein concentration in the samples.

Activities of four key antioxidant enzymes in the liver, namely GPx, GR, CAT and SOD, were measured using commercial kits in triplicate (Nanjing Jiancheng Bioengineering Institute, Nanjing, China). For GPx, the enzymatic activity was measured by the rate of formation of oxidized glutathione (GSSG). Homogenized liver samples (5 µL) were incubated with 5, 5′-dithiobis (2-nitrobenzoic acid) (DTNB at 100 µL) to generate TNB for 10 min, and the concentration of TNB was measured afterwards as described above. Positive controls were performed by adding 20 µmol/L GSH solution in 5 µL. The activity of GPx was calculated following the manufacturer’s protocol and expressed as U/mg protein. 

The GR activity was determined by measuring the rate of NADPH oxidation [28]. A small amount (10 µL) of homogenized liver sample was added to 200 µL of 1 mM NADPH solution. The concentration of oxidized NADPH was measured by absorbance at 340 nm for 3 min. Each unit of GR activity is defined as the amount of protein that oxidized 1 mM NADPH per min. 

The activity of a CAT was determined by the catalyzed decomposition of a fixed concentration of hydrogen peroxide (1 μmol; H_2_O_2_) per min. Firstly, 10 µL of homogenized liver samples were incubated with ammonium molybdate (220 μL) for 10 min. The residual ammonium molybdate was reacted with H_2_O_2_ to generate a yellowish complex (OD at 240 nm). One unit of catalase activity is defined as 1 µmol H_2_O_2_ consumed per mg of protein per sec. The activity of SOD was measured based on the inhibition of tetrazolium salt (WST-1) reduction. SOD reduces the natural oxidation of WST-1, which would convert it into a water-soluble formazan dye. Liver homogenates (20 µL) were incubated with WST-1 solution (200 µL) for 20 min, and SOD activity was calculated based on formazan dye concentrations measured by absorbance at 450 nm. One unit of SOD activity is defined as the amount of enzymes required to inhibit the oxidation reaction by 50% and was expressed as U/mg protein.

### 2.6. Serum Collection and Proteomic Sample Preparation

After the bacterial challenge, zebrafish serum was collected from healthy and disease conditions following a published protocol [29]. Fish was euthanized by immersion into 1.0 mg/mL MS-222 (Sigma, Burlington, MA, USA) for 2 min. The caudal peduncle of each zebrafish was then cut with a scalpel. Fish, with wounds pointing down, were individually placed into 0.5 mL microcentrifuge tubes with a small hole at the end. This 0.5-mL microcentrifuge tube containing the fish was, in turn, placed inside a 1.5 mL microcentrifuge tube (Eppendorf, Hamburg, Germany). This assembly was centrifuged at 700 rpm for 5 min at 12 °C to collect blood. As each zebrafish could only give small amounts of blood, we pooled blood from 5 individuals as one replicate. Blood was allowed to clot on ice for 30 min, and serum was separated from blood cells by centrifugation at 1500 rpm for 10 min at 4 °C. Serum was stored at 80 °C until analysis. Three replicates of serum samples were collected for each treatment. Protein concentrations of each serum sample was determined using BCA protein assay (Thermo Scientific, Waltham, MA, USA).

From each replicate, 10 µg serum protein was then diluted with 5 µL of 6 M urea buffer (6 M urea, 50 mM dithiothreitol (DTT), 10 mM Tris-HCL, pH 8.0, Sigma, Burlington, MA, USA) and then reduced with 5 µL of 5 mM DTT in 25 mM ammonium bicarbonate for 45 min at 56 °C, and then underwent alkylation with 10 µL of 14 mM iodoacetamide (IAA, Sigma, Burlington, MA, USA) in 25 mM ammonium bicarbonate for 30 min at room temperature in the dark. 

The protein in each sample was allowed to precipitate using cold acetone for 2 h at −20 °C. Centrifugation at 14,000 rpm for 10 min at 4 °C was used to harvest the protein and the protein pellet was air-dried for 30 min. The protein pellet was then dissolved in 2 µL of 6 M urea buffer containing 1 µg trypsin (Promega, Madison, WI, USA) and 20 µL of 25 mM ammonium bicarbonate at 37 °C overnight. Digestion was stopped by acidification (pH < 3) using 5% trifluoroacetic acid (TFA, Sigma, Burlington, MA, USA) the following morning. Peptides were then extracted using C18 ZipTip (Millipore, Burlington, MA, USA) pre-equilibrated with 0.1% TFA. After sample loading, peptides were eluted with 80% acetonitrile (ACN, MS grade, Sigma, Burlington, MA, USA) in 0.1% TFA. Then, CentriVap Centrifugal Vacuum Concentrators (Labconco, Kansas City, MO, USA) were used to dry the peptides, which were resuspended in 10 µL of 0.1% formic acid (FA, Sigma, Burlington, MA, USA) afterwards. 

For each sample, 1 µL of 1 fmol/µL typically digested alcohol dehydrogenase protein from *Saccharomyces cerevisiae* (Sigma, Burlington, MA, USA) was added as an internal standard prior to LC-MS/MS analysis. Each unique peptide concentration detected by LC-MS/MS was normalized to the concentration of the internal standard peptides [30].

### 2.7. Serum Proteomics Analysis

Peptide samples were analyzed by ultra-high-performance liquid chromatography (UHPLC, UltiMate 3000 Rapid Separation Quaternary System, Thermo Scientific, Waltham, MA, USA) coupled with an Orbitrap Fusion Lumos Tribrid Mass Spectrometer (Thermo Scientific, Waltham, MA, USA). For each sample, 2 µg of digested serum containing the internal standard was loaded in a nanoflow C18 column (15 cm in length, 75 µm in diameter; Thermo Scientific, Waltham, MA, USA) equilibrated in 0.1% FA. Peptides were eluted with a linear gradient of 5–30% solvent B (80% ACN in 0.01% TFA) at a constant flow rate of 300 nL/min for over 120 min. Eluted peptides were analyzed with an Orbitrap tandem mass spectrometer in positive mode using the following settings: spray voltage 2800 V and temperature of the capillary 300 °C. The maximal injection time was 20 ms. Full-scan MS spectra were acquired from 350 to 1500 *m*/*z*, with a resolution of 60,000 and an automatic gain control (AGC) target of 400,000. DDA mode was used to acquire the data. For MS/MS scans, the activation type was set to higher-energy collisional dissociation (HCD) with a collision energy of 30%. The Orbitrap MS/MS scan range mode was set on auto (*m*/*z* Normal) with a resolution of 15,000, and the AGC target was set as 50,000. Ions were injected using all available parallelizable times, and the maximum injection time was 30 ms.

Data collected were searched against the National Center for Biotechnology Information nonredundant (NCBInr) database containing all zebrafish protein entries (143,725 sequences; 8 November 2011), and the NCBInr database with a taxonomy filter of *Saccharomyces cerevisiae* using Mascot v. 2.3.2 (Matrix Science, Chicago, IL, USA). The following settings were used: ‘trypsin’ was selected for the enzymes used, ‘Carbamidomethyl (C)’ and ‘Oxidation (M)’ were selected for variable modifications, the peptide mass tolerance was set at 20 ppm, the peptide charges were 2+, 3+, and 4+, the mass tolerance for MS/MS was set at 0.1 Da. Any search results with ion scores of less than 20 were discarded [31]. The false discovery rate (FDR) was set at 1%.

For label-free quantitation, proteins with a ≤0.80- or ≥1.20-fold difference were regarded as differentially expressed proteins (DEPs) as previous studied [32]. DEPs were identified using MaxQuant v. 1.2.2.5 on DDA raw files in each sample, as described by Cox et al. [33]. Database acquisition and quantitative parameters were the same as above. Normalized intensity values (LFQ intensity) were calculated and exported for quantitation using Perseus v. 1.6.0.2. DEPs were obtained from two pairs of comparisons, namely between (1) healthy animals of SeNP and control diets and between (2) disease animals of SeNP and control diets. 

Zebrafish serum proteomic results were imported into the Ingenuity Pathway Analysis (IPA, Version 01–13, Ingenuity Systems, Redwood City, CA, USA) and Database for Annotation, Visualization and Integrated Discovery (DAVID) v6.8 to explore gene ontology (GO) functional annotations. Enrichment scores of proteins were expressed as −log10 (*p*-value) to assess the probability that annotations in the protein list could be due to chance, assuming an underlying hypergeometric distribution. Canonical pathway and molecular network analyses were determined using Ingenuity Pathway Analysis (IPA) core analysis (Version 01–13, Ingenuity Systems, Redwood City, CA, USA) using DEPs. Matched proteins encoding genes from the Ingenuity Knowledge Base were used to generate molecular networks to understand the biological and molecular functions of DEPs. Right-tailed Fisher’s exact tests were utilized to determine the probability that biological functions and/or diseases were associated with particular proteins. Fisher’s exact test was used to determine the statistical significance and canonical pathways with enrichment scores of 2 or higher were considered as significantly enriched pathways. 

### 2.8. Transcriptome Analysis

The transcriptome analysis of livers and kidneys was carried out to make two pairs of comparisons between (1) healthy animals of SeNP and control diets and between (2) disease animals of SeNP and control diets. The total RNA of the kidneys and livers were extracted with the RNeasy Mini kit (Qiagen, Hilden, Germany), according to the manufacturer’s instructions. Purity of the RNA samples was determined with a NanoDrop 200 spectrophotometer (Thermo Scientific, Waltham, MA, USA), quality of the samples was determined with the Agilent 2100 Bioanalyzer (Agilent Technologies, Santa Clara, CA, USA) and concentration of RNA samples were measured by the Agilent RNA 6000 Nano Kit (Agilent Technologies, Santa Clara, CA, USA). All RNA samples used for RNA-seq have OD260/280 = 1.8~2.2, OD260/230 = 1.8~2.2, without degradation and contamination, and RNA integrity number (RIN) value ≥7.0. 

Sequencing library construction and Illumina sequencing were performed. Firstly, mRNA was enriched via purification with oligo (dT) magnetic beads and broken into short fragments in a fragmentation buffer [34]. First-stand cDNA was then synthesized using random hexamer-primed reverse transcription using fragmented mRNA as the templates. Following that, complementary second-strand cDNA was synthesized using DNA polymerase I. Then, the double-stranded cDNA was purified by magnetic beads and sequencing, and adaptors were ligated to the terminal end of the purified cDNA. Next, these products were subjected to TAE-agarose and further enriched using PCR amplification. Enriched fragments were again purified by magnetic beads and dissolved in the appropriate amount of Epstein–Barr solution and sequenced using an Illumina HiSeq 4000 platform.

Sequence raw reads were generated with the Sequence Platform. Data filtering is carried out to obtain high-quality reads as the clean reads (clean data) and filter out adaptor sequences and/or low-quality reads present in the raw reads. The quality of sequencing was presented high, as the ratio of clean reads in raw reads was over 98% for all samples. The clean reads were mapped to a reference genome of *Danio rerio* GRCz downloaded from Ensembl (ftp://ftp.ensembl.org, accessed on 7 April 2022) using hierarchical indexing for the spliced alignment of transcripts (HISAT2). Clean reads data were imported into the HTSeq Python package to map the reads to genes. The fragment per kb per million fragments (FPKM) method was used in calculating the expression level. DESeq2 was used to identify differential expressed genes (DEGs) defined as log fold change values larger than 1 and *p*-values less than 0.01 [35,36].

Transcriptome profiles were integrated, and the extent of overlap was presented in Venn diagrams (http://bioinformatics.psb.ugent.be/webtools/Venn/, accessed on 7 April 2022). A cluster analysis of DEGs was carried out using an R package heatmap. For function analysis, GOseq (version: v1.16.2) was used to compare the DEGs to reference genes for GO enrichment analysis. Web Gene Ontology Annotation Plot (WEGO) was used for visualizing, comparing and plotting the GO annotation results (corrected *p*-value < 0.01) [37]. KEGG was used to perform a pathway enrichment analysis of the DEGs. In addition, a scatter plot was used to display the RichFactor, *p*-value and DEG number of the enriched pathways. RichFactor is the ratio of differentially expressed gene numbers annotated in a pathway compared to all gene numbers annotated in the pathway.

### 2.9. ICP-MS Analysis

Tissue samples (including brain, gill, gut, kidney, liver, gonad and muscle) were collected by necropsy to determine the total Se concentration. Tissues were weighed (mg dry weight) and transferred into 50 mL digestion tubes containing 1 mL 70% nitric acid (metal grade, Sigma, Burlington, MA, USA). Digestion was carried out in heat block for 2 h at 95 °C and 0.25 mL 30% H_2_O_2_ (Sigma, Burlington, MA, USA) to the tube and continued to be heated for another 2 h at 95 °C. The digested mixture then was diluted four-fold with 5% nitric acid and filtered through 0.45-µm Hydrophilic Teflon filters (Sigma, Burlington, MA, USA). Filtered samples were furthered diluted five-fold with 5% nitric acid in 15-mL Falcon tubes to achieve a final volume of 5 mL. Indium was added as an internal standard for determination of consistency of machine efficiency. ICP-MS (Agilent 7500, Santa Clara, CA, USA) was used to determine the total Se concentration. Using spiked samples and certified reference materials (DORM-4, National Research Council Canada, Ottawa, ONT, Canada), the average Se recovery of our method was 92.4%. MilliQ water was used as blank sample.

### 2.10. Statistical Analyses

Survivorship of fish in these treatments was compared using a Kaplan–Meier analysis coupled with log-rank test (Mantel–Cox) and Gehan–Breslow–Wilcoxon tests in SPSS (ver 15.0, IBM SPSS Statistics, Chicago, IL, USA). Other endpoints were compared using one-way ANOVA with Duncan’s multiple comparison and two-way ANOVA using both diet and disease as fixed factor with SPSS. All data were presented as mean ± SD and considered to be significantly different at the *p* < 0.05 level.

## 3. Results

### 3.1. SeNPs and SeNP Diet Characterization

**Figure 1:**
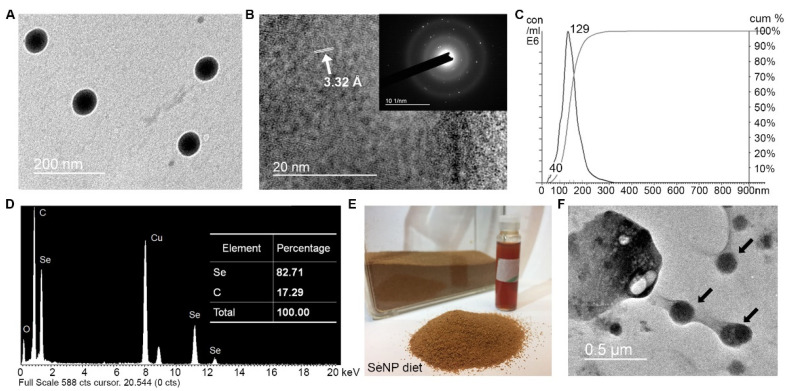
Characterization of chitosan-stabilized SeNPs and the diet: (**A**) representative TEM image of SeNPs; (**B**) representative HR-TEM image of SeNPs (Inset: SAEN pattern of SeNPs); (**C**) size distribution of SeNPs by NanoSight (Mean: 129 nm, SD: 40 nm); (**D**) Elemental analysis by EDX spectrum; (**E**) representative image of SeNP diet; (**F**) representative TEM image of SeNP diet (black arrows indicated SeNPs attaching on fish diet particulates).

Chitosan-stabilized SeNPs are largely spherical, with an average diameter of 67.08 nm (SD = 4.84 nm) and haves a homogeneous structure (Figure 1A). High-resolution transmission electron microscopy (HRTEM) images show a clear lattice d-spacing value of 3.32 Å (Figure 1B). Selected Area Electron Diffraction (SAED) suggested that this SeNP has a polycrystalline structure, which is similar to other polysaccharide-stabilized SeNPs produced using a similar method [4,5]. The stability of the nanomaterials is critical for its application as a nutrient supplement; thus, we measured the size distribution and stability of SeNPs with a mean size at 129 nm and standard deviation (SD) at 40 nm (Figure 1C). An Energy-Dispersive X-ray Analysis (EDX) indicated that SeNPs contained mostly selenium (Se, 82.71%) and some carbon (C, 17.29%), which was likely from the chitosan surface stabilizer (Figure 1D). Copper (Cu) peaks were visible in the EDX spectra due to the Cu support grid. No other obvious peaks for the other elements were observed in EDX, confirming that the SeNPs were of high purity.

As shown in Figure 1E,F, the SeNP diet had a proper size for adult zebrafish, and the SeNPs were well-attached to fish feed particulates under TEM observation. These spherical NP-like structures are not shown in the control diet. From ICP-MS, the control diet has 3.6 μg/g Se, and the SeNP diet has 13.2 μg/g Se. The results in the control and SeNP diet were similar to the value reported by earlier studies using the same base fish diet [3,5,38].

### 3.2. Comparison of Immunomodulation Effects with SeNP and Trolox Diet

**Figure 2:**
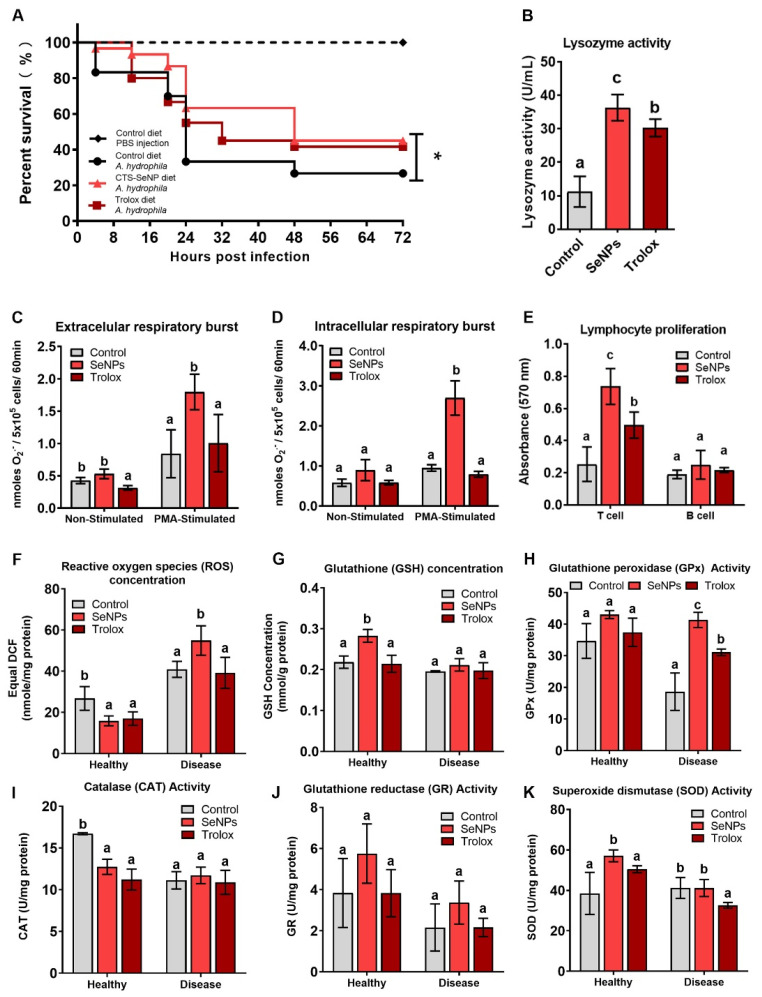
Dietary SeNPs and Trolox-induced immune and antioxidant responses: (**A**) Bacterial challenge by Aeromonas hydrophila in the zebrafish fed with SeNP, Trolox or control diets; (**B**) Lysozyme activity; (**C**) Extracellular respiratory burst activity; (**D**) Intracellular respiratory burst activity; (**E**) Lymphocyte proliferation; (**F**) ROS concentration; (**G**) GSH concentration; (**H**) GPx activity; (**I**) CAT activity; (**J**) GR activity; (**K**) SOD activity. Different alphabets in the figure indicate statistically different groups where * *p* < 0.05.

There was no mortality in PBS-injected zebrafish. For (A) hydrophila-injected fish, individuals displayed symptoms, including abdominal swelling, hemorrhagic septicemia and mortality. The survival rate (A) of hydrophila-injected fish fed the control diet decreased to 70% after 24 h and significantly decreased to 33.3% at 48 h, finally reaching 26.7% after 72 h (Figure 2A). Although both the SeNP and Trolox diets had protective effects on zebrafish against (A) hydrophila infection, the SeNP diet showed more protection at 24 h post-injection, with an 86.7% survival rate compared with 66.7% in the Trolox group. After 72 h post-injection, for the SeNP diet group, the survival rate was 45.0%. For the Trolox diet group, the effect was similar, and the survival rate was 41.7% after 72 h.

Despite having equal antioxidant capacity, the Trolox and SeNP diets had different effects on the immune system. The serum lysozyme activity was significantly increased by both the SeNP and Trolox diets, but SeNP had a significantly higher effect than Trolox (Figure 2B). Both diets had little effect on the phagocytic respiratory burst without a stimulation (Figure 2C,D). When stimulated by PMA, however, only the SeNP diet led to a significantly higher response than the control. 

The two diets also had different effects on immune cell proliferation. Both diets had no significant effect on B-cell proliferation. However, both diets had a significant effect on T-cell proliferation, but SeNP had significantly higher effect than Trolox (Figure 2E).

### 3.3. Comparison of Antioxidation Effects with SeNP and Trolox Diet

Both the SeNP and Trolox diets led to a significant decrease of the total ROS concentration in healthy fish by 40.7% and 36.6%, respectively (Figure 2F). Fish with disease had a significant increase in the total ROS when compared with healthy fish. Trolox had no effect on the ROS concentration in diseased fish, while SeNP led to a significantly more pronounced increase when compared with the control. Two-way ANOVA also showed significant interactions between the diet and different health conditions (*p* < 0.01).

A reduced GSH concentration was significantly increased by SeNP in both healthy and disease conditions (Figure 2G). However, the magnitude of the increase was higher in healthy conditions than in disease conditions, and this was confirmed by the significant interaction of feeding treatments and different conditions in two-way ANOVA (*p* < 0.05). Trolox had no significant effect on reduced GSH concentrations in both conditions. 

SeNP and Trolox diets again had different effects on activities on the four tested enzymes. For GPx and GR, their activities decreased in diseased individuals when compared with healthy individuals. The SeNP diet improved their activities in both healthy and diseased conditions. For GPx, the SeNP diet could eliminate the decrease completely in diseased individuals (Figure 2H). Trolox had no difference in GPx activity in healthy individuals but led to a smaller decrease in diseased individuals. Trolox had no significant effect on GR activity when compared with the control (Figure 2I).

For CAT, a decrease in activity was observed when comparing individuals with disease against healthy individuals (Figure 2J). Both the SeNP and Trolox diets significantly decreased CAT activity in healthy individuals but had no effect on individuals with the disease. The SeNP and Trolox diets significantly increased SOD activity in healthy fish (Figure 2K). In diseased individuals, SeNP had no significant effect, but Trolox significantly decreased SOD activity.

### 3.4. Serum Proteomic Analysis

**Figure 3:**
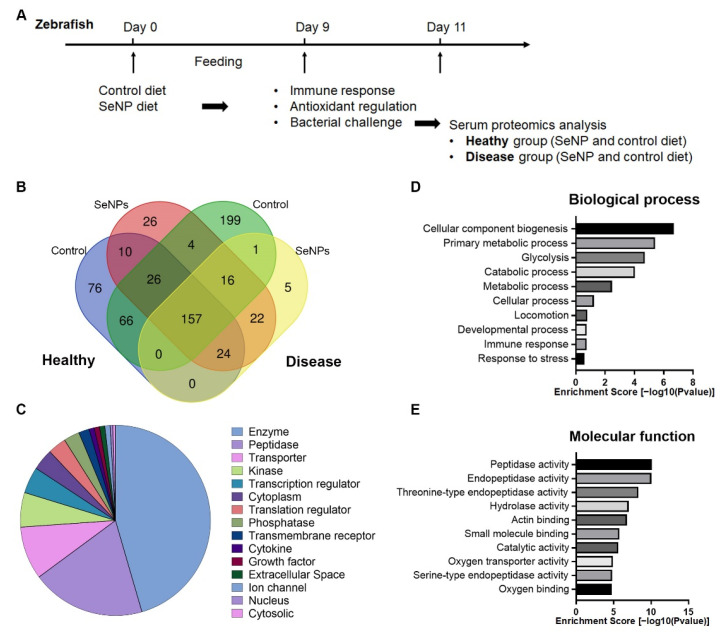
Serum proteomic analysis with/without dietary SeNPs in healthy and diseased conditions: (**A**) Experimental scheme of serum proteomic analysis; (**B**) Venn diagram depicting the total number of identified protein families in zebrafish serum from the control diet and SeNP diet in healthy conditions and control diet and SeNP diet in diseased condition. (**C**) Protein type category by IPA classification; (**D**) GO term biological processes analysis of serum proteins; (**E**) GO term molecular functions analysis of serum proteins.

To examine serum protein regulation by SeNP, we collected serum samples from healthy and disease zebrafish fed with the SeNP diet and control diets for 9 days (Figure 3A). A total of 632 protein families were identified in the zebrafish serum samples, including healthy and diseased conditions and 157 protein families were common to all groups (Figure 3B). GO functional annotation in the IPA reported that most of serum proteins were classified as enzymes (45.5%), peptidases (19.4%) and transporters (9.0%) (Figure 3C). DAVID classified the serum proteins into three main categories (biological processes, molecular functions and cellular components). For biological processes, cellular component organization or biogenesis had largest enrichment score (6.68), followed by primary metabolic process (5.41), glycolysis (4.70) and catabolic process (4.03) (Figure 3D). For molecular functions, all of the top categories were enzyme activities such as peptide activity (10.11), endopeptidase activity (10.06), threonine-type endopeptidase activity (8.30) and hydrolase activity (7.01) (Figure 3E). The number of identified protein families and serum protein classification was all comparable with the reported zebrafish plasma proteomic profiles [29,39].

We identified a total of 190 differentially expressed proteins (DEPs) between the control and SeNP diets in healthy conditions and 185 DEPs under diseased conditions (details in Supplementary Table S1). In a healthy condition, parvalbumin isoform X1 (XP_005164345) was decreased 41.67-fold in the SeNP diet. Jeltraxin (XP_001331789) and carboxylesterase 3 precursor (NP_001038401) were increased in the SeNP diet with 29.24-fold and 10.93-folds, respectively. In disease conditions, pyruvate kinase isozyme M1/M2 isoform X1 (XP_005163170) was the most differentially expressed with a 14.21-fold decrease in the SeNP diet treatment. Complement C4-B (XP_001334640) and immunoglobulin light-chain constant region (ABC59635) were the most increased in expression with 13.57-fold and 13.50-fold change, respectively.

### 3.5. Canonical Pathway and Molecular Network Analyses from Serum Proteomics

**Figure 4:**
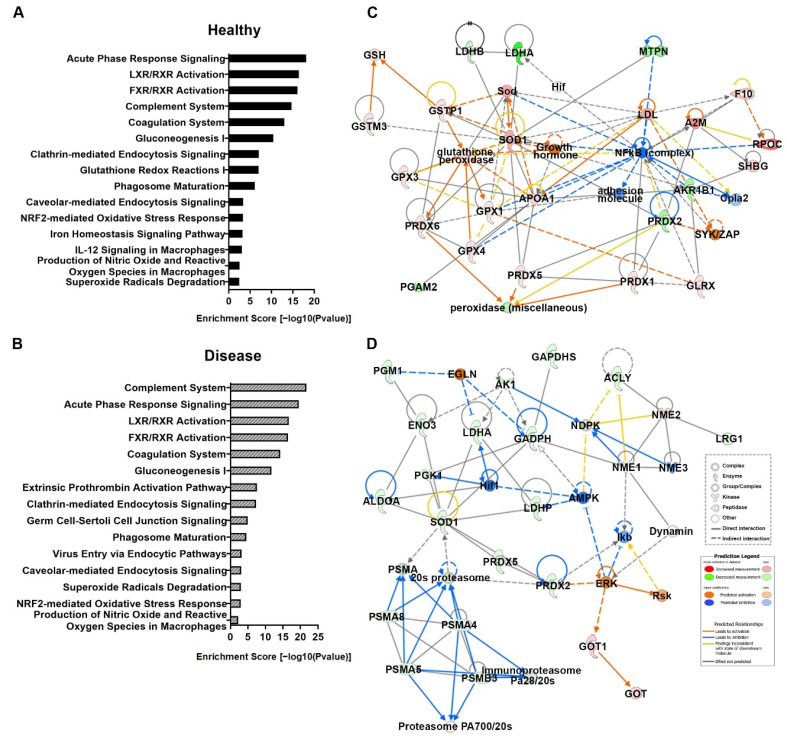
Top 15 enriched canonical pathways identified in IPA by DEPs in (**A**) healthy and (**B**) diseased conditions. The pathways are indicated on the y-axis. On the x-axis, the enrichment score (−log10(*p*-value)) for each pathway is indicated by the bars. (**C**) Healthy top molecular network involving free radical scavenging, small molecule biochemistry and drug metabolism. (**D**) Disease top molecular network in conditions involving carbohydrate metabolism, nucleic acid metabolism, small molecule biochemistry. Upregulated proteins are represented in red and downregulated proteins are represented in green. Orange represents predicted activation, while blue represents predicted inhibition.

Enriched canonical pathways and molecular networks in IPA were employed using the DEPs between the SeNP and control diets in healthy and diseased conditions. In canonical pathways, an analysis of the 45 total enriched pathways were identified in healthy conditions, and 46 enriched pathways were identified in diseased conditions. The top 15 significantly enriched pathways in the healthy and diseased conditions were largely identical and were related to immune and antioxidant activities (Figure 4A,B).

The top 5 enriched pathways in both conditions were all related to immune regulation. In healthy conditions, the most significantly enriched pathways were the acute phase response signaling pathway (Figure 4A, Enrichment score 18.10), followed by LXR/RXR activation (Enrichment score 16.40), FXR/RXR activation (Enrichment score 16.10), complement system (Enrichment score 14.70) and coagulation system pathways (Enrichment score 13.00). In diseased conditions, the most significantly enriched pathways were the complement system pathway (Figure 4B, Enrichment score 21.70), followed by the acute phase response signaling pathway (Enrichment score 19.50), LXR/RXR activation (Enrichment score 16.70), FXR/RXR activation (Enrichment score 16.40) and coagulation system pathways (Enrichment score 14.20).

Molecular network analyses identified a total of nine networks that were significantly enriched in the healthy condition and 11 networks in the diseased condition. In the healthy condition, the top three enriched molecular networks were related to free radical scavenging, lipid metabolism and immunological disease (Table 1). In the diseased condition, the top three enriched molecular networks were related to carbohydrate metabolism, lipid metabolism and immunological disease (Table 1). In all these molecular networks, about half of the proteins in the networks were also identified as DEPs in our previous analysis. Interestingly, two out of the three top molecular networks under healthy and diseased conditions were identical (Lipid metabolism and Immunological disease). For a Lipid metabolism molecular network, the DEPs involved were almost identical in both conditions (18 out of 21), suggesting that the role of SeNP on lipid metabolism remained unchanged in the healthy or diseased conditions. This contrasted the other common molecular network (Immunological disease), where only a few DEPs involved were common to both conditions (7 out of 20), suggesting that the role of SeNPs on the immune system was different in the healthy and diseased conditions.

In healthy conditions, the top enriched molecular network was related to free radical scavenging. Interactions of the molecules revolve around two important molecule hubs of SOD and NFkβ (complex) (Figure 4C). In the diseased conditions, the top enriched molecular network was carbohydrate metabolism. Interactions of the molecules showed that there were two important molecule groups, and they communicate via modulation of SOD1 (Figure 4D).

### 3.6. Uptake of Se and Transcriptomic Response of Kidney and Liver

**Figure 5:**
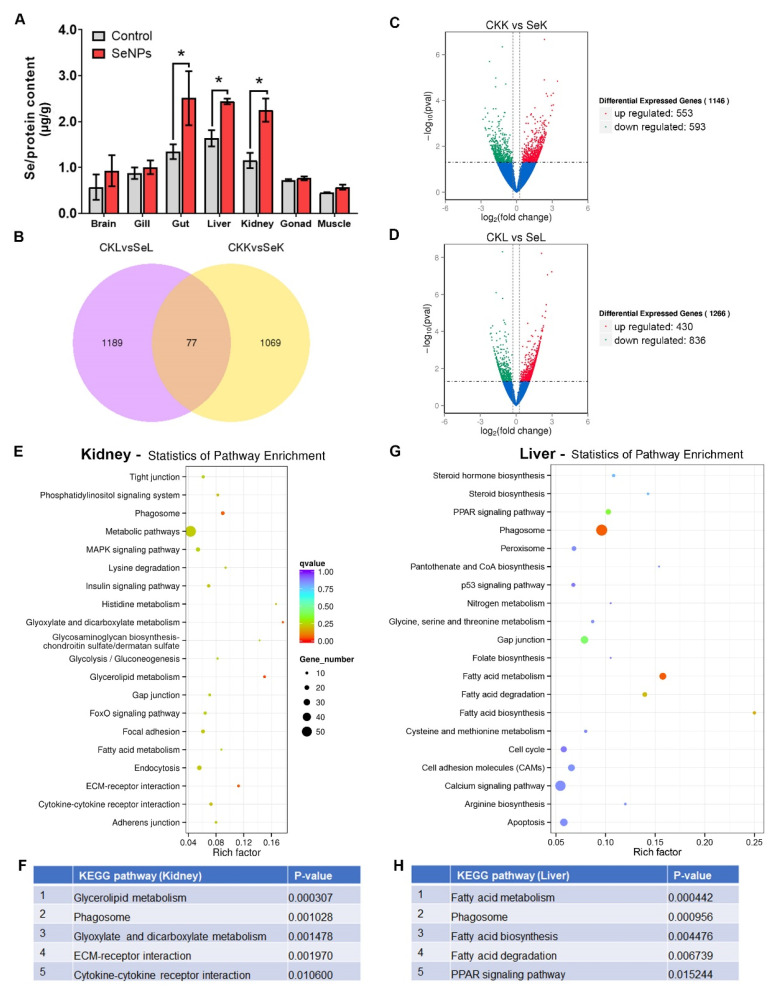
Transcriptome analysis of Se-enriched tissues (liver and kidney): (**A**) Se concentration in seven tissues, including the brain, gill, gut, liver, kidney, gonad and muscle; (**B**) Venn diagram of DEGs in the liver and kidneys; (**C**) Volcano plots of kidney DEGs; (**D**) Volcano plots of liver DEGs; (**E**) Kidney DEG-enriched KEGG pathway; (**F**) Top 5 KEGG-enriched pathways in kidneys; (**G**) Liver DEG-enriched KEGG pathway; (**H**) Top 5 KEGG enriched pathway in the liver. * *p* < 0.05.

The uptake of Se was only significant in the gut (1.34 to 2.51 µg/g), liver (1.63 to 2.44 µg/g) and kidneys (1.15 to 2.25 µg/g) after the 9-day exposure period (Figure 5A). This finding is consistent with previous studies that the liver and kidneys are the most sensitive tissues in dietary Se supplementation [24]. We finally decided to perform RNA-Seq analysis on the liver and kidneys instead of the gut because of two reasons: (1) liver and kidneys are metabolism and immune direct-related tissues with markedly elevated protein-bound Se accumulation and (2) the variation in SeNPs gut samples implies the diet residue contributes to the Se level inside the gut. 

Overall, we found 1266 differentially expressed genes (DEGs) in the liver and 1146 DEGs in the kidneys comparing the control and SeNPs diet groups (Figure 5B, details in Supplementary Table S2). As shown in the volcano plot, there were 836 upregulated DEGs and 430 downregulated DEGs in the kidneys after dietary SeNPs (Figure 5C). There were 553 upregulated DEGs and 593 downregulated DEGs in the liver (Figure 5D). The Venn diagram showed that only 77 DEGs were common in both tissues (Figure 5B). Through inputting the DEGs into the KEGG pathway, and enriched pathways in the kidneys and liver tissues were identified (Figure 5E,G). The top five enriched KEGG pathways in the kidneys by dietary SeNPs includes ECM–receptor interactions, cytokine–cytokine receptor interactions, glyoxylate and dicarboxylate metabolism, glycerolipid metabolism and phagosome (Figure 5F). Biological functions of these top five enriched KEGG pathways are related with NP uptake (such as ECM–receptor interactions, cytokine–cytokine receptor interactions and phagosomes) and energy metabolism (glyoxylate and dicarboxylate metabolism and glycerolipid metabolism). 

In the liver, the top five enriched KEGG pathways includes fatty acid metabolism, phagosome, fatty acid biosynthesis, fatty acid degradation and the PPAR signaling pathway (Figure 5H). Biological functions of these top five enriched KEGG pathways are also related with NP uptake (such as phagosome) and energy metabolism (fatty acid metabolism, fatty acid biosynthesis, fatty acid degradation and PPAR signaling). One of the DEGs related to the fatty acid metabolism pathways (such as fatty acid metabolism, fatty acid biosynthesis and fatty acid degradation) was acaca (acetyl-CoA carboxylase alpha, ENSDARG00000078512) with a 1.44-fold changed decrease in the SeNP group compared to the control. Two acyl-CoA synthetase-related genes (acsbg2 and acsl3b) increased 0.51- and 0.79-fold changed expression by SeNP.

## 4. Discussion

SeNPs offered a protective effect against *A. hydrophilla* infection, resulting in slower and fewer deaths from the disease. The protective effect of SeNP against bacterial infection is supported by our previous study with the same SeNP5 and studies with other SeNPs [40]. Our findings are similar to previous reports of other SeNPs showing an immunomodulation effect, such as SeNPs protected mice from 7,12-dimethylbenz(a)anthracene (DMBA)-induced immunotoxicity and increased the number of leucocytes [41]. Our previous study also demonstrated that SeNPs could increase immune responses, such as lysozyme activity, phagocytic respiratory burst, and lymphocytes proliferation [5]. However, the underlying mechanism is not yet understood.

Although the immunomodulation effect of SeNP appeared to relate to its antioxidant activities, its effect was different from the exogenous antioxidant Trolox. Trolox showed similar effects to SeNP on lysozyme activity and T-cell proliferation but were less effective. Trolox also had no effect on intracellular and extracellular respiratory bursts. Interestingly, both SeNP and Trolox offered similar magnitudes of protective effects the *A. hydrophilla* challenge, suggesting that the increase in lysozyme activity alone may be sufficient to counter bacterial infection. Regarding antioxidant regulation, SeNP also has positive effects on more biomarkers than Trolox. In particular, SeNP is positive on the glutathione system, while Trolox is not. Since key enzymes GPx and GR are both selenoenzymes, the positive effect of SeNP on their activity might suggest that the animal is able to metabolize the SeNP and utilize the Se to make more selenoproteins. If this is true, then SeNP will likely have positive effects on other selenoprotein-related bioactivities. This should be verified in future research.

In this study, zebrafish serum samples were analyzed in healthy and diseased conditions with or without SeNP diet supplementation. Serum proteomics and Se-enriched tissues of transcriptomics provide opportunities to understand the systemic response to SeNP. Based on the canonical pathways and molecular network analysis by IPA, the enriched pathways and molecular networks confirmed that SeNPs exerted biological functions through immune system regulation and antioxidant activities. 

The top biological functions of SeNPs were consistent in healthy and diseased individuals (Figure 3). The top canonical pathways in healthy and disease individuals were related to immune functions (e.g., acute phase response signaling and complement system), redox regulation (e.g., superoxide radicals degeneration) and pathways that were shown to relate to nanoparticle exposure and uptake (e.g., endocytosis). Similar results were observed in the molecular networks where the top three molecular networks in both conditions were related to immune functions, lipid metabolism and redox regulations (Table 1). 

The immunomodulation activity of SeNPs was observed under healthy conditions. The top canonical pathway based on the DEP analysis was acute phase immune responses. Acute phase response signaling is a prominent systemic reaction of the organism through regulating serum concentrations of a class of proteins to local or systemic disturbances caused by infection, tissue injury, trauma or surgery, neoplastic growth or immunological disorders [42]. This is in agreement with the upregulation of a large number of complement proteins (Table 1). The second and third canonical pathways (LXR/RXR activation and FXR/RXR activation, respectively) are also related to acute phase responses. Studies on human rectal mucosa showed that the Se status altered the inflammatory signaling and cancer risk by the inhibition of LXR/RXR and FXR/RXR activation pathways [43]. While an improved acute phase response could help the individual to respond to a pathogen assault, it may also indicate inflammation. However, a molecular network analysis showed that the NFkβ cascade is suppressed, suggesting that SeNP also has anti-inflammatory properties [44].

Under disease conditions, SeNPs modulated the complement system pathway. The complement system represents an efficient first line of defense against microbial infection [45]. IPA predicted the upregulation of C3 and C1q, which, in turn, would initiate activation of the complement system via the alternative pathway and classical pathway, respectively. The Se status was reported to relate to expression of the key factor of complement system C3 [46]. Activation of the complement system resulted in the upregulation of downstream C5b, C6, C7 and C8 to trigger a membrane attack complex synthesis. We also recorded the upregulation of the C6, C7 and C8 proteins, confirming the prediction of IPA. The molecular network analysis also showed an increase of the ERK cascade expression and decrease in the SOD expression. ERK is closely associated with NFkβ and the release of other proinflammatory cytokines, suggesting that SeNP increased the inflammatory response under diseased conditions. 

The immunomodulation activity of chitosan SeNP could be closely related to its antioxidant activity, as shown by the top canonical pathways. This link was also demonstrated on other Se species in a number of studies [47]. The antioxidant activity of Se was associated with recovering GPx activity, decreasing ROS-mediated lipid peroxidation and regenerating the GSH [48]. In healthy individuals, the top molecular network of SeNP treatment was related to free radical scavenging. This was confirmed by measurements of biomarkers related to redox regulation in the individual. GPx and GR activities and the GSH concentration in the liver were increased by the SeNP diet in both healthy and diseased conditions. Such changes could improve the immune functions, as GSH was required for the proliferation of cells (lymphocytes and interstitial epithelial cells) and activation of T cells and polymorphonuclear leukocytes in vivo [49]. Similarly, we observed an increased leukocyte count and improved T-cell activation. The glutathione system is one of the most important antioxidant systems against ROS, with the central antioxidant compound GSH [47].

Interestingly, SeNP caused the ROS concentration to decrease in healthy individuals but increase in diseased individuals. A lower level of ROS in healthy individuals should be considered beneficial, as a high ROS level was reported to relate to a number of chronic diseases in humans. An overproduction of ROS can result in oxidative damage to biomolecules such as lipids, proteins and DNA, which has been implicated in the development of aging, as well as various ailments, including cancer, respiratory, cardiovascular, neurodegenerative and digestive diseases [50]. In healthy individuals, enriched canonical pathways related to ROS regulation include glutathione redox reactions I, superoxide radicals degradation, NRF2-mediated oxidative stress response, Iron homeostasis signaling and the production of nitric oxide and ROS in macrophage pathways. The top enriched molecular network also predicted the activation of a number of key enzymes related to ROS regulation, including SOD1, GPx1, 3 and 4, and various peroxidases. In the antioxidant system, SOD plays an important role in catalyzing the dismutation of the superoxide radical (O_2_^−^) into either ordinary molecular oxygen (O_2_) or hydrogen peroxide (H_2_O_2_) and protects the cellular components from being oxidized by ROS [51]. Many studies have also reported that SeNPs increased the antioxidant defense, resulting in a decrease of ROS generation by studying healthy individuals [52].

In diseased individuals, SeNP led to a higher ROS concentration in the animal when compared to those fed with normal fish feed. There are two possible explanations: (1) increase in the injury associated with ROS increase and/or (2) increase in inflammation and macrophage activity. It was reported that the upregulation of ROS generation and phagocytosis during sepsis and bacterial infection [53] activated phagocytes that would release ROS during a respiratory burst as an immune response to eliminate foreign particles and bacteria to combat infections [5]. Ristow reviewed evidence of ROS benefits and suggested that a hormesis effect is observed for ROS, where some level of ROS is beneficial to health [54]. In the light of SeNP-protected individuals against a bacterial challenge, we argue that this increase in ROS concentration under the disease was beneficial. The downregulation of 20 S proteasome and a number of proteasome subunit alpha types (PSMA) (Supplementary Information Table S1) is also suggestive of a lower number of bacteria-infected cells in SeNP-treated individuals. release of proteasomes has been suggested as a mechanism through which components derived from intracellular pathogens may be presented to the immune system, and their protein content can be modified under pathological or stress conditions [47]. Previous publications suggest that extracellular vesicles released from cells infected with intracellular bacterial pathogens contain bacterial components [55]. A recent study also reported that oseltamivir surface-modified SeNPs increased ROS generation to inhibit the activity of influenza virus glycoproteins, thus preventing the H1N1 virus from infecting kidney cells [56]. This new report supported our finding that SeNPs could have a dual role in the regulation of ROS in the host, depending on their health condition. Such regulation is likely through the up- and downregulation of SOD1 and NFκB cascades. Under healthy conditions, SOD1 was upregulated while NFκB was downregulated, and the opposite was observed under the diseased condition. 

The transcriptome analysis showed that the phagosome pathway is enriched in both the kidneys and liver after supplied with SeNPs (Figure 5). Compared with inorganic and organic Se, the nano-scale elemental Se particles can easily enter the cells and translocate across the cell, tissues and organs to exert their function [57]. Phagocytosis is one of the active forms of endocytosis. During this process, the phagocytes, including monocytes, macrophages and neutrophils, will stimulate the formation of a membrane-bound vesicle called a phagosome to ingest the target material such as antigen or external materials into the cells [58]. The natural positive charged chitosan cannot pass through the hydrophobic plasma membrane; therefore, SeNPs are internalized by an active transportation called endocytosis [59]. It implies that the chitosan-stabilized the SeNP can effectively enter multiple tissues and exert its Se supplementary functions. 

Further, according to our proteomic analysis, the transcriptome analysis also showed that gene expressions in fatty acid metabolism are modulated by SeNPs. A previous study also reported that Se supplementation induced ROS modulation of lipid synthesis in a pig liver [27]. In the KEGG fatty acid metabolism pathway, acetyl-CoA carboxylase alpha (acaca) was downregulated (1.44-fold change) in the SeNP liver. This suppressed the activity of acetyl-CoA carboxylase (ACC) through phosphorylation, which can reduce the inflammatory reaction to further protect innate immune cells [21]. This implies that lipid metabolism can play an important role as a ‘buffer’ in SeNP immunomodulation against over-inflammation-induced immune cell impairment. Taken together, the transcriptome analysis suggested SeNP uptake was primarily through active phagosome transportation in Se metabolic tissues and provided an explanation on how lipid metabolism neutralizes the inflammatory response in immune cells. This finding echoes with serum proteomic profiles and supports our hypotheses, which is that SeNPs modulate the immune system in different directions, depending on the condition of the host. In the healthy condition, uptaken SeNPs decrease ROS generation to inhibit inflammation and reduce oxidative stress, while, in the diseased condition, SeNPs rapidly mobilize redox metabolisms to increase in inflammation and macrophage activity.

## 5. Conclusions

In conclusion, chitosan-stabilized SeNPs showed benefits to an individual beyond the effects of an antioxidant. It showed more broad-spectrum effects on the immune system than exogenous antioxidants such as Trolox, and SeNPs have a positive effect on the glutathione system via the promotion of activities and possibly even synthesis of selenoenzymes. We studied the mechanism of immunomodulation of the activities of chitosan-stabilized SeNPs under healthy and diseased conditions. We used a proteomic analysis on the serum and transcriptome analysis on the kidneys and liver to identify molecular pathways and biological functions SeNPs can influence under healthy and diseased conditions. Antioxidant regulation, lipid metabolism and innate immune responses were modulated in both healthy and diseased individuals. These activities appeared to be intertwined to produce the observed immunomodulation benefits, especially important in rapid switching in inflammatory responses between healthy and diseased conditions. Our transcriptomic profiles in Se-enriched tissues (kidneys and the liver) showed that SeNP provides immune responses that induce the energy requirements via lipid metabolism behaviors. Overall, this study supports that chitosan-stabilized SeNPs have great potential in immunomodulation function through systematic biological regulations, including antioxidation, inflammation and lipid metabolism.

## Figures and Tables

**Table 1:** The top three molecular networks based on differentially expressed proteins (DEPs) comparing the two diets under healthy and diseased conditions, respectively. DEPs are indicated in bold text.

Analysis	ID	Molecules in Network	Score	Focus Molecules	Top Diseases and Functions
**Healthy** (SeNP diet vs. Control diet)	1	**A2M**, adhesion molecule, **AKR1B1**, **APOA1**, Cg, Cpla2, **F10**, **GGCT**, **GLRX**, glutathione peroxidase, **GPX1**, **GPX3**, **GPX4**, Growth hormone, GST, **GSTM3**, **GSTP1**, Hif, Ldh (complex), **LDHA**, **LDHB**, LDL, **MTPN**, NFkβ (complex), peroxidase (miscellaneous), **PGAM2**, **PRDX1**, **PRDX2**, **PRDX5**, **PRDX6**, **PROC**, **SHBG**, Sod, SOD1, SYK/ZAP	45	22	Free Radical Scavenging, Small Molecule Biochemistry, Drug Metabolism
	2	**AHSG**, Alpha 1 antitrypsin, **APOA4**, **APOB**, **BHMT**, Ces, **CES1**, **CETP**, **CFH**, **CP**, creatine kinase, ERK1/2, **ESD**, Ferritin, **FGA**, **GC**, HDL, HDL-cholesterol, hemoglobin, **HPX**, **ITIH4**, **ITLN1**, MHC Class II (complex), Nos, Nr1h, PRKAA, **RBP4**, **SERPINA1**, **SERPINA9**, **SERPINC1**, **SERPINF2**, **SH3BGRL**, **TF**, VLDL, VLDL-cholesterol	43	21	Lipid Metabolism, Small Molecule Biochemistry, Vitamin and Mineral Metabolism
	3	Akt, C3, C5, C6, C7, C8, C1q, C3-Cfb, C5-C6-C7, C5-C6-C7-C8, C5-C6-C7-C8-C9, **C8A**, **C8B**, **C8G**, **CFI**, Collagen Alpha1, Collagen type IV, Collagen(s), Complement, Complement component 1, **CSTB**, elastase, Fibrin, Fibrinogen, **HABP2**, **HBE1**, **HBZ**, Kallikrein, Laminin (complex), LRG1, MAC, **NME3**, Pdi, **PLG**, **SERPING1**	30	16	Immunological Disease, Developmental Disorder, Hereditary Disorder
**Disease** (SeNP diet vs. Control diet)	1	20s proteasome, **ACLY**, **AK1**, **ALDOA**, AMPK, Dynamin, EGLN, ENO3, ERK, **GAPDH**, **GAPDHS**, GOT, GOT1, Hif1, Ikb, Immunoproteasome Pa28/20s, **LDHA**, **LDHB**, **LRG1**, NDPK, **NME1**, **NME2**, **NME3**, **PGK1**, **PGM1**, **PRDX2**, **PRDX5**, Proteasome PA700/20s, PSMA, **PSMA4**, **PSMA5**, **PSMA8**, **PSMB3**, Rsk, **SOD1**	44	22	Carbohydrate Metabolism, Nucleic Acid Metabolism, Small Molecule Biochemistry
	2	**AHSG**, Alpha 1 antitrypsin, **AMBP**, **APOA4**, **APOB**, **CES1**, **CETP**, **CFH**, **CP**, creatine kinase, ERK1/2, Ferritin, **FGA**, **GC**, **HBZ**, HDL, HDL-cholesterol, hemoglobin, **HPX**, Iti, **ITIH3**, **ITIH4**, **ITLN1**, MHC Class II (complex), Nos, Nr1h, PRKAA, **RBP4**, **SERPINA1**, **SERPINA9**, **SERPINC1**, **SERPINF2**, **TF**, VLDL, VLDL-cholesterol	41	21	Lipid Metabolism, Small Molecule Biochemistry, Vitamin and Mineral Metabolism
	3	**A2M**, **C6**, **C7**, C3-Cfb, C5-C6-C7, C5-C6-C7-C8, C5-C6-C7-C8-C9, **C8A**, **C8B**, **CAPNS1**, **CFB**, **CFD**, **CFI**, **CFP**, chymotrypsin, coagulation factor, **CORO1A**, Ecm, elastase, **F9**, **F10**, glutathione peroxidase, Hif, Kallikrein, Ldh (complex), **MASP2**, NFkβ (complex), **PAPSS2**, **PLG**, **PROC**, **PRSS2**, Serine Protease, **SERPING1**, **SOD3**, trypsin	38	20	Immunological Disease, Developmental Disorder, Hereditary Disorder

## Data Availability

Data is contained within the article and supplementary material.

## Supplementary Materials

The following supporting information can be downloaded at https://www.mdpi.com/article/10.3390/antiox11050964/s1: Table S1: Differentially expressed proteins in healthy and disease condition; Table S2: Differentially expressed genes in healthy and disease condition.
